# TkMYB7 Coordinates Jasmonate and Ethylene Signaling to Regulate Natural Rubber Biosynthesis in *Taraxacum kok-saghyz*

**DOI:** 10.3390/plants14213323

**Published:** 2025-10-30

**Authors:** Xiaodong Li, Yulin Wu, Changping Zhang, Gaoquan Dong, Lin Xu, Yuya Geng, Zihan Guo, Yan Zhang, Jie Yan

**Affiliations:** 1 Key Laboratory of Xinjiang Phytomedicine Resource and Utilization of Ministry of Education, College of Life Sciences, Shihezi University, Shihezi 832003, China; xiaodongli202407@163.com (X.L.); yulinwu2023@163.com (Y.W.); 15009357340@163.com (C.Z.);; 2 Xinjiang Production and Construction Corps Key Laboratory of Oasis Town and Mountain-Basin System Ecology, College of Life Sciences, Shihezi University, Shihezi 832003, China; 3 College of Agricultural, Shihezi University, Shihezi 832003, China; 4 Crop Research Institute of Xinjiang Uygur Autonomous Region Academy of Agricultural Sciences, Urumqi 830091, China

**Keywords:** *Taraxacum kok-saghyz*, MYB transcription factors, TkMYB7, yeast one-hybrid, MVA pathway, *Arabidopsis thaliana*

## Abstract

Russian dandelion (*Taraxacum kok-saghyz* Rodin, TKS) is a natural rubber (NR)-producing species whose roots contain 3% to 27% NR, underscoring its considerable research and economic significance. The myeloblastosis (MYB) transcription factor family, one of the largest in plants, plays pivotal roles in metabolic regulation, stress responses, and various growth and developmental processes. To identify key MYB transcription factors involved in hormone-induced rubber biosynthesis, we conducted homology-based and bioinformatic analyses to characterize 268 MYB family proteins in the TKS genome. Utilizing transcriptome data from jasmonic acid (JA) and ethylene (ET) treatments, we screened and shortlisted 10 candidate *TkMYB* transcription factors. Through tissue-specific expression profiling, *TkMYB7* was selected as the primary candidate. We confirmed that promoter analysis combined with yeast one-hybrid assays confirmed that *TkMYB7* directly binds to and regulates the expression of acetyl-CoA acetyltransferase (*TkACAT5*), a key enzyme in the mevalonate (MVA) pathway. Furthermore, heterologous overexpression of *TkMYB7* in *Arabidopsis thaliana* significantly enhanced seed germination and root development. These findings identify *TkMYB7* as a novel transcriptional regulator linking JA and ET signaling pathways to rubber biosynthesis in TKS, representing a promising target for the genetic improvement of rubber yield.

## 1. Introduction

Russian dandelion (*Taraxacum kok-saghyz* Rodin, TKS), a perennial herbaceous plant belonging to the Asteraceae family, has garnered significant attention due to its ability to produce natural rubber (NR) from its roots [1,2]. The natural rubber (cis-1,4-polyisoprene) extracted from TKS roots is an invaluable polymer characterized by outstanding physical properties, including elasticity, abrasion resistance, and extensibility, which collectively render it a critical raw material in industries such as aerospace, transportation, and medicine [3]. However, as global economic development progresses, the primary source of NR, *Hevea brasiliensis*, is increasingly unable to meet the rising demand for this material. Its cultivation faces challenges due to geographical limitations, extended growth cycles, and vulnerability to viral infections [4,5]. In contrast, TKS presents several advantages, including ease of cultivation, simplified harvesting processes, shorter harvest cycles, and high-quality rubber production [2,6]. Given these agronomic advantages, research has increasingly focused on elucidating the fundamental biological processes—particularly the hormonal regulation—that govern natural rubber biosynthesis in TKS.

Plant hormones are endogenous chemical messengers that regulate a wide array of physiological processes, including growth, development, root formation, flowering, and fruiting, typically exerting their effects at very low concentrations. Among these hormones, jasmonic acid (JA) and its derivatives—such as methyl jasmonate and jasmonoyl-isoleucine—are collectively referred to as jasmonates (JAs). JAs play pivotal roles not only in governing fundamental aspects of plant growth and development but also in modulating responses to both biotic and abiotic stresses [7]. Notably, exogenous application of JA has been shown to significantly enhance plant tolerance to various abiotic stresses, including drought and temperature extremes. This effect has been validated in multiple species such as *Arabidopsis thaliana*, *Triticum aestivum*, and *Zea mays*, where JA treatment positively contributes to improved drought resistance [8,9,10]. Beyond its general role in stress adaptation, jasmonic acid (JA) has emerged as a key master regulator of a specific and economically important process: the synthesis of plant secondary metabolites.

Furthermore, studies have demonstrated that jasmonic acid (JA) is intricately involved in the synthesis of plant secondary metabolites [11]. Among these metabolites, natural rubber represents a crucial secondary compound in rubber-producing plants, and the regulatory interplay between JA and rubber synthesis has been a focal point of extensive research. Previous investigations have particularly emphasized the promotion of laticifer differentiation; for instance, wounding or stripping the young bark of *Hevea brasiliensis* has shown that exogenous JA application can lead to an increase in laticifer number [12,13,14,15]. The expression levels of JA-responsive genes, such as *COI1*, *MYB*, *HB8*, and *ARF8*, exhibit a remarkably similar trend to the increase in laticifers within the secondary phloem tissue [14], providing compelling evidence that JA acts as a regulator of laticifer differentiation. With advancements in biotechnology, an increasing body of research has established that transcription factors (TFs) within the JA signaling pathway serve as key regulators of NR synthesis. In rubber trees, JA regulates NR biosynthesis through a comprehensive signaling cascade (COI1-JAZ3-MYC2), wherein this complex binds to specific sequences in the promoters of rubber synthesis-related genes, thereby modulating NR synthesis [16,17]. Additionally, other studies have indicated that MYB TFs in Hevea can also interact with the promoters of rubber biosynthesis genes such as *HTR1* and *SRPP* [18,19], further suggesting that MYB TFs play significant roles in the NR synthesis pathway. Indeed, the regulatory roles of MYB transcription factors are increasingly supported by recent functional genomics studies in rubber-producing plants.

Natural rubber biosynthesis is tightly controlled by plant hormone signaling, with jasmonate (JA) and ethylene (ET) recognized as two of the most pivotal activators [1,2]. In *Hevea brasiliensis*, JA not only induces laticifer differentiation but also modulates core components of its signaling pathway, such as MYC transcription factors, which are directly regulated by MYB proteins. For example, a recent genome-wide investigation identified HbMYB88, which directly binds to the promoter region of the *HbMYC-1* gene within the JA signaling cascade, thereby serving as a crucial link in laticifer cell differentiation and rubber biosynthesis [20]. Similarly, in Russian dandelion (*Taraxacum kok-saghyz*, TKS), the MYB-related protein TkMYBR090 is specifically expressed in latex and is proposed to function through the modulation of JA precursor metabolism [21]. Collectively, these findings strongly indicate that MYB transcription factors act as key downstream nodes of JA and ET signaling pathways, orchestrating the regulation of natural rubber biosynthesis. To fully appreciate the centrality of MYB TFs, it is essential to consider the context of ethylene signaling, another major hormonal pathway converging on NR production.

Ethylene, another essential plant hormone, is widely involved in various physiological processes, including fruit ripening, leaf senescence, and root growth. It plays a unique role in modulating plant secondary metabolism [22]. As early as the 1970s, studies revealed that the application of ethephon to the bark of rubber trees stimulates latex regeneration and enhances rubber yield [23], indirectly demonstrating that ethylene can promote natural rubber production. Recent multi-omics studies suggest that the increased yield mediated by ethylene represents a stress defense response; ethylene serves as a defense signal that upregulates the expression of genes encoding latex proteins [24,25,26]. Furthermore, ethylene does not operate in isolation; it synergizes with other plant hormones. Through such synergistic interactions, ethylene and other hormones activate transcription factors (TFs) involved in amino acid biosynthesis and energy metabolism pathways, thus providing the requisite enzymatic proteins and energy compounds necessary for NR synthesis [24,27,28]. The convergence of JA and ET signaling on transcriptional regulators brings the role of specific TF families, particularly the MYB family, into sharp focus.

Transcription factors (TFs) are pivotal molecules in the regulation of gene expression, exercising control by binding to specific DNA sequences, recruiting additional proteins, or altering chromatin structure. The MYB family represents one of the most significant TF families in plants, playing crucial roles in responses to biotic and abiotic stresses, as well as in development, differentiation, metabolism, and defense mechanisms [29]. MYB TFs are characterized by a highly conserved N-terminal DNA-binding domain, known as the MYB domain, which specifically binds to consensus sequences (e.g., C/TAACG/TG and ACCA/TAA/CT/C). The C-terminal region typically contains multiple conserved motifs within subfamilies that may function as activation or repression domains during transcriptional regulation [30,31]. The MYB family is categorized based on the number of MYB domain repeats, including 1R-MYB, R2R3-MYB, R1R2R3-MYB, and 4R-MYB, with a less common 5R-MYB type also identified. Among these classifications, R2R3-MYB constitutes the largest and most functionally significant subfamily in plants. Additionally, 1R-MYB proteins serve as vital telomere-binding proteins, essential for maintaining chromosomal structural integrity and stability, and hold substantial biological significance in cell and tissue morphogenesis as well as circadian rhythms [30,31,32].

MYB transcription factors (TFs) are essential for the formation of various plant organs, with extensive studies focusing on their role in root development. MYB proteins can either promote or inhibit root growth, responding to environmental stresses and participating in phytohormone signaling pathways to regulate both primary and lateral root development. In Arabidopsis, MYB44 has been shown to be involved in the auxin signaling pathway, promoting primary root growth, while MYB73 and MYB77 also modulate lateral root development through auxin regulation. Conversely, MYB93 has been identified as an inhibitor of lateral root development [33,34,35]. Parallel to their roles in development, MYB TFs are equally vital for controlling the biosynthesis of secondary metabolites.

MYB transcription factors (TFs) play pivotal roles in the regulation of secondary metabolite biosynthesis. For instance, CsMYB42 enhances the expression of *CsGS1c*, thereby promoting the accumulation of theanine, whereas CsMOF1 acts as a negative regulator by repressing *CsGS1* activity. In *Malus domestica*, MdMYB85 facilitates the late-stage production of ester-derived aromas by upregulating the expression of the biosynthesis-associated gene alcohol acyltransferase 1 (*MdAAT1*) [36,37,38]. Emerging evidence further indicates that MYB TFs contribute to plant adaptive responses to both biotic and abiotic stresses through the modulation of secondary metabolism. For example, in *Populus tomentosa*, PtoMYB142 directly binds to the promoters of wax biosynthesis genes (*CER4* and *KCS6*), thereby promoting leaf wax deposition to mitigate drought-induced water loss [39]. This established capacity of MYB TFs to regulate diverse metabolic pathways logically extends to the complex biochemical pathway of natural rubber synthesis.

The synthesis of natural rubber (NR) is fundamentally centered on the production of isopentenyl pyrophosphate (IPP), which serves as the key precursor for subsequent rubber chain elongation. IPP is predominantly synthesized via two distinct pathways: the mevalonate (MVA) pathway, which is the primary route for rubber biosynthesis, and the methylerythritol phosphate (MEP) pathway, operating within the cytoplasm and plastids, respectively [40,41]. The MVA pathway utilizes sucrose as a substrate, which is first hydrolyzed by invertases into glucose and fructose. Subsequently, glucose is subjected to glycolysis and a series of enzymatic reactions that yield acetyl-CoA, a crucial substrate implicated in various aspects of plant secondary metabolism [4,40,41]. Acetyl-CoA is then converted into IPP through a succession of six enzymatic steps, facilitated by key enzymes including acetoacetyl-CoA thiolase (ACAT), 3-hydroxy-3-methylglutaryl-CoA synthase (HMGS), 3-hydroxy-3-methylglutaryl-CoA reductase (HMGR), mevalonate kinase (MVK), phosphomevalonate kinase (PMVK), and mevalonate diphosphate decarboxylase (MVD) [4]. Furthermore, earlier studies have indicated that MYB transcription factors (TFs) can interact with critical components of the rubber biosynthesis pathway, such as small rubber particle protein (*SRPP*) and rubber transferase (*HTR1*), thereby participating in the regulation of NR synthesis [4,18,19]. This underscores the multifunctional role of MYB TFs in the regulation of secondary metabolites.

However, in TKS, a promising alternative rubber-producing species, the specific R2R3-MYB members responsible for perceiving JA and ET signals and directly regulating the rubber biosynthesis pathway remain unidentified. Against this background, our study proposed a clear hypothesis: in TKS, MYB transcription factors responsive to JA and ET signaling are the most probable key regulators of natural rubber biosynthesis. To test this hypothesis, a systematic screening approach was employed. First, transcriptome data from TKS roots treated with JA and ET were analyzed to identify candidate genes among the 268 annotated TkMYB members that were co-upregulated and significantly induced by both hormones. This approach aimed to pinpoint candidates directly linked to known hormonal activators of rubber biosynthesis. Through this screening, the candidates were narrowed to ten. Among these, *TkMYB7* was selected as the primary focus due to its notably high and root-specific expression, consistent with roots being the principal site of rubber synthesis. This study thus aims to detail the identification of TkMYB7 and experimentally validate its function as a transcription factor that directly regulates the key mevalonate (MVA) pathway enzyme gene *TkACAT5*, thereby elucidating its pivotal role within the transcriptional regulatory network of rubber biosynthesis in TKS.

## 2. Results

### 2.1. Identification and Phylogenetic Analysis of the TkMYB Gene Family

**Figure 1:**
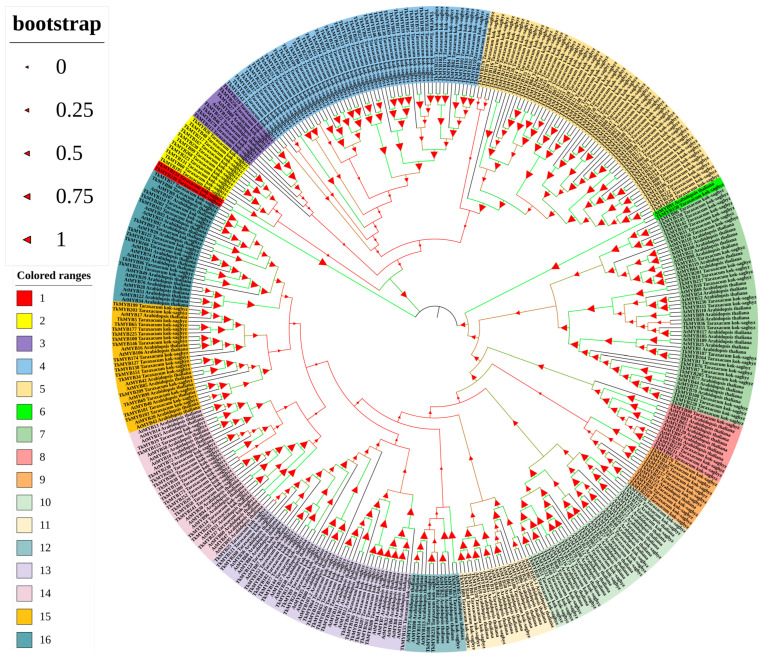
Phylogenetic tree of *TkMYB* genes constructed from the *Taraxacum kok-saghyz* (TKS) genome in comparison with *Araidopsis thaliana*. Colored blocks denote distinct subgroups, numbered 1 to 16. Bootstrap values are shown on the branches to indicate the confidence level of each clade.

A total of 268 non-redundant R2R3-MYB transcription factors were identified in the *Taraxacum kok-saghyz* (TKS) genome (for details of the identification procedure, see Section 4). Phylogenetic analysis of these proteins, together with 126 MYBs from *Arabidopsis thaliana*, revealed that the 394 transcription factors are grouped into four major clades and 16 distinct subgroups (Figure 1). This classification highlights the substantial diversity and potential functional specialization of the MYB family in TKS, comparable to that observed in *Arabidopsis*. The reliability of the phylogenetic relationships is supported by high bootstrap values in the majority of branches. Moreover, TKS MYB transcription factors exhibited strong sequence homology with their *Arabidopsis* counterparts. For instance, the close phylogenetic proximity between *TkMYB150* and *AtMYB91* suggests potential conservation of function between these proteins (Figure 1).

### 2.2. Transcriptome Analysis of Ethylene and Methyl Jasmonate Responses in the TkMYB Gene Family

**Figure 2:**
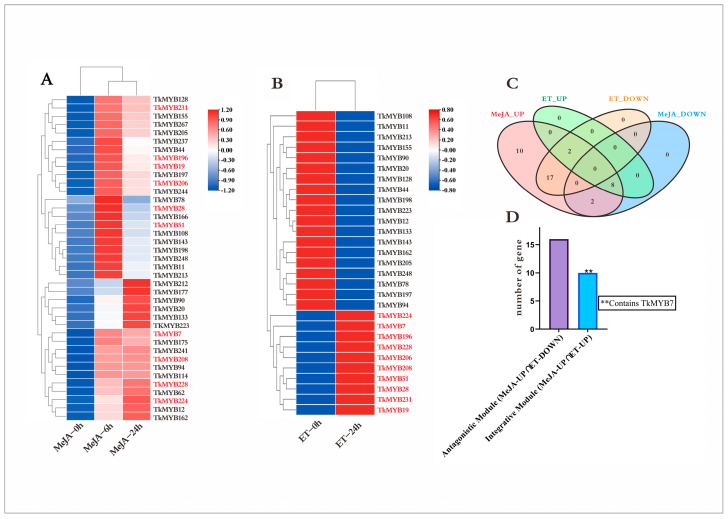
Transcriptomic responses of TKS MYB transcription factors to MeJA and ET treatments: (**A**) Heatmap showing the expression dynamics of 39 MeJA-responsive MYB TFs at 0, 6, and 24 h. Colors represent relative expression levels (blue, lower; red, higher); scale bars indicate normalized expression values. (**B**) Heatmap of 29 ET-responsive MYB TFs at 0 and 24 h, with upregulated and downregulated patterns visible across samples; (colors as in panel (**A**)). (**C**) Venn diagram summarizing the overlap between MeJA-upregulated (MeJA-UP), MeJA-downregulated (MeJA-DOWN), ET-upregulated (ET-UP), and ET-downregulated (ET-DOWN) MYB TFs. All ET-responsive TFs are contained within the MeJA-responsive set, with 10 TFs upregulated by both hormones and 19 showing antagonistic regulation (MeJA-UP ∩ ET-DOWN). (**D**) Bar chart quantifying the two functional modules: the antagonistic module (MeJA-UP ∩ ET-DOWN; 19 genes) and the integrative module (MeJA-UP ∩ ET-UP; 10 genes). The integrative module includes TkMYB7, as indicated.

Plant hormones play pivotal roles in regulating growth and development. To examine the responsiveness of MYB transcription factors (TFs) to hormonal stimuli, *Taraxacum kok-saghyz* (TKS) plants were subjected to treatments with both methyl jasmonate (MeJA) and ethylene (ET). Transcriptomic datasets derived from MeJA- and ET-treated samples were analyzed to determine differential MYB TF expression. The results indicated that 39 MYB TFs were responsive to MeJA (Figure 2A), whereas 29 MYB TFs responded to ET (Figure 2B). Remarkably, all 39 MeJA-responsive MYB genes displayed an overall upregulation trend, while 10 out of the 29 ET-responsive genes were upregulated. To pinpoint TFs responsive to both hormones, a Venn diagram analysis was conducted. As shown in Figure 2C, the entire set of 29 ET-responsive TFs was encompassed within the MeJA-responsive TF set. Given that plant hormones often interact in synergistic or antagonistic manners, these TFs were further categorized into functional modules (Figure 2D), with 19 genes assigned to the antagonistic module and 10 genes assigned to the synergistic module. This selection criterion was adopted because jasmonate (JA) and ethylene (ET) are well-established inducers of natural rubber biosynthesis. We reasoned that MYB transcription factors co-induced by both pathways are high-priority candidates for coordinating the rubber biosynthetic network. Accordingly, the 10 TFs in the integrative set were prioritized for downstream analyses, with a particular focus on assessing root-enriched expression—the primary site of rubber production in TKS.

Notably, the sampling time points for transcriptome analysis were determined based on the distinct signaling kinetics of each hormone, with the aim of detecting regulators that exhibit stable upregulation. For MeJA treatment, samples were collected at 0, 6, and 24 h to capture both its rapid and sustained transcriptional responses, as extensively documented in our previous transcriptomic and promoter analyses [5,42]. For ET treatment, samples were collected at 0 and 24 h to focus on its stable induction of metabolic regulators—a characteristic frequently reported in hormone-induced rubber biosynthesis studies [24,27].

### 2.3. Analysis of Conserved Domains, Gene Structure, and Protein Physicochemical Properties of TkMYB Family Members

**Figure 3:**
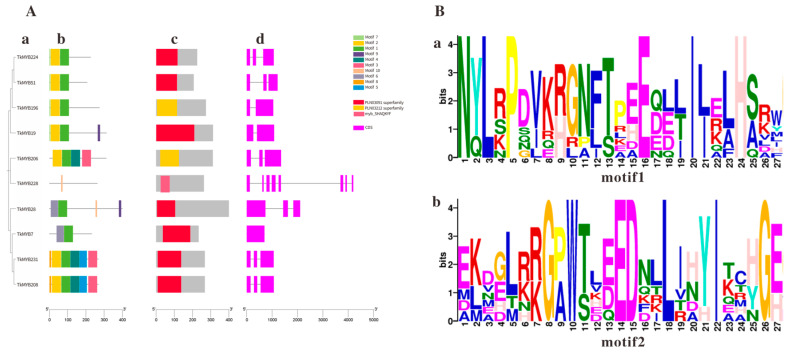
Phylogenetic tree, motif composition, and conserved domain analysis within the MYB family in the TKS genome: (**A**) (**a**) Phylogenetic tree of TkMYB family members. (**b**) Motif composition of TkMYBs. (**c**) Conserved domains of TkMYB family members. (**d**) Exon/intron information for TkMYB family genes; purple rectangles represent exons, black lines represent introns. (**B**) Sequence logos; the *X*-axis indicates sequence position within the conserved Motif, the *Y*-axis represents the degree of sequence conservation at each position. (**a**) Conserved motif motif1; (**b**) Conserved motif motif2.

Analyses of evolutionary relationships, protein structures, and gene structures were conducted on the 10 MYB genes identified. The results demonstrated that phylogenetic tree analysis divided the 10 TkMYB proteins into 7 subgroups, Figure 3A(a). Conserved motifs and domains of TkMYB proteins were identified using the MEME motif online search engine and the NCBI CD-Search domain prediction tool, Figure 3A(b,c). The exon-intron organization of the TkMYB genes was analyzed using the Gene Structure Display Server tool Figure 3A(d). A total of ten conserved motifs were identified among the 10 TkMYB proteins Figure 3A(b), which were consistent with the predicted structures; nearly all MYB genes contained the conserved Motif 1 and Motif 2 (Figure 3B). Multiple sequence alignment revealed that Motif 1 and Motif 2 correspond to the core segments of the R2 and R3 repeats, which collectively form the canonical DNA-binding domain of R2R3-MYB transcription factors [30,43]. The high conservation of these motifs across most candidates strongly suggests that they function through specific DNA binding, as demonstrated for TkMYB7. Notably, the absence of Motif 2 in TkMYB228 suggests it may lack a functional DNA-binding domain, potentially indicating a divergent regulatory role, such as acting as a dominant-negative regulator or having acquired a novel, non-canonical function [44]. Furthermore, the phylogenetic analysis of the TkMYB family indicated that proteins within the same subgroup exhibited similar exon-intron structures Figure 3A(d), suggesting that proteins within the same clade often share analogous biological functions.

Physicochemical properties of the 10 identified MYB proteins were analyzed (Table 1). The length of TkMYB amino acid sequences ranged from 207 (TkMYB51) to 400 (TkMYB28), molecular weight from 24.35 kDa (TkMYB51) to 45.05 kDa (TkMYB28), predicted isoelectric point (pI) from 5.77 (TkMYB206) to 9.97 (TkMYB28), and instability index from 46.51 (TkMYB228) to 65.70 (TkMYB51), indicating these are all unstable proteins. The lower instability index limit (46.51 for TkMYB228) is significantly higher than the threshold of 40, suggesting potential for rapid dynamic regulation. Subcellular localization predictions indicated that all TkMYB proteins are localized in the nucleus. GRAVY values ranged from −1.114 (TkMYB51) to −0.578 (TkMYB28), indicating all proteins are hydrophilic.

### 2.4. Cloning and Subcellular Localization Analysis of TkMYB7

**Figure 4:**
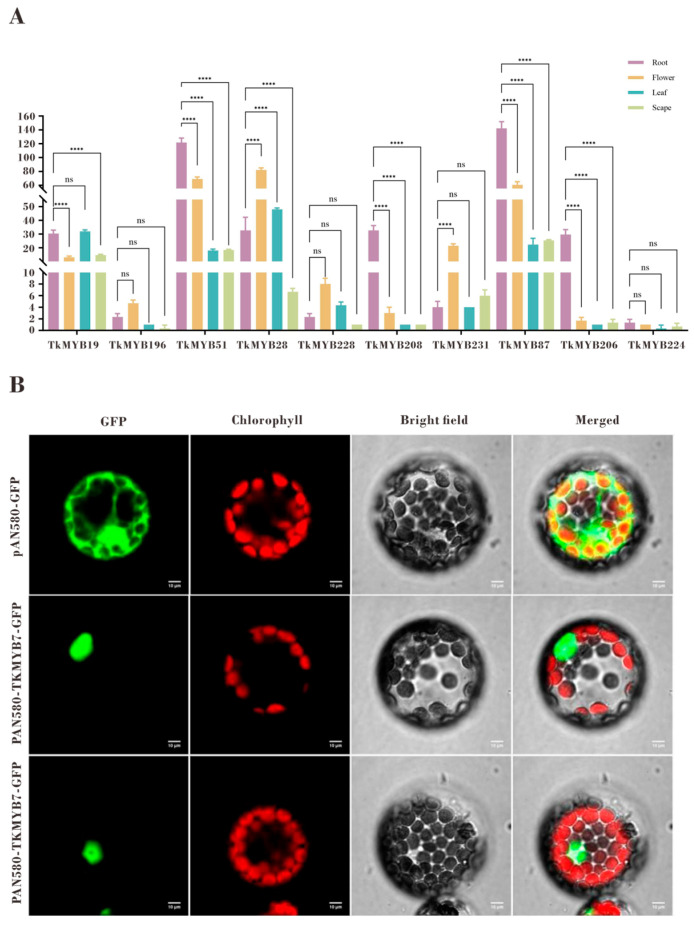
Tissue-specific expression patterns and subcellular localization of TkMYB7 and other candidate *Taraxacum kok-saghyz* MYB transcription factors: (**A**) Quantitative PCR (qPCR) analysis of the ten integrative-module MYB candidates in four different tissues: root, flower, leaf, and scape. Expression levels were normalized against reference genes, and statistical significance was determined by one-way ANOVA with post-hoc testing (**** *p* < 0.0001; ns, not significant). (**B**) Subcellular localization of TkMYB7 in *Arabidopsis protoplasts*. GFP fluorescence driven by the pAN580 control vector was distributed throughout the cytoplasm and nucleus (top row), whereas TkMYB7–GFP fusion protein localized predominantly to the nucleus (middle and bottom rows). Chlorophyll autofluorescence (red) marks chloroplasts. The merged images demonstrate nuclear GFP signal distinct from chloroplast localization, confirming nuclear targeting of *TkMYB7*. Scale bars = 10 μm.

Tissue-specific expression profiling by quantitative PCR (qPCR) revealed distinct expression patterns among the ten candidate MYB genes (Figure 4A). The qPCR results were consistent with the transcriptome data and demonstrated that TkMYB7 exhibited markedly higher expression in roots compared with other candidates. Phylogenetic analysis (Figure 1, subfamily 7, highlighted in cyan) showed that TkMYB7 clustered closely with AtMYB44 and AtMYB77, two Arabidopsis homologs known to participate in root development and auxin signaling [33,34]. The combination of predominant root-specific expression and its evolutionary association with MYB proteins involved in root growth identifies TkMYB7 as the most promising candidate regulator.

Subcellular localization analysis further supported its potential regulatory role. Prediction indicated nuclear localization (Table 1), which was experimentally validated by GFP-fusion protein assays in Arabidopsis protoplasts. The GFP signal co-localized with the nuclear marker, confirming nuclear localization of TkMYB7 (Figure 4B). These findings collectively highlight TkMYB7 as a high-priority target for functional assays to elucidate its role in both rubber biosynthesis and root development.

### 2.5. Transcriptome and Promoter Analysis of MVA Pathway Genes

**Figure 5:**
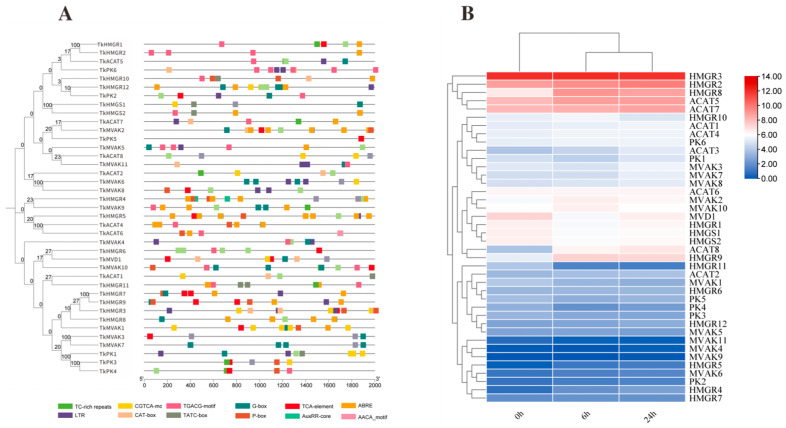
Promoter element prediction and expression levels after JA treatment for key enzyme genes in the TKS MVA pathway: (**A**) Promoter element prediction for TKS MVA pathway genes; each color represents a type of element. (**B**) Expression levels of key MVA pathway enzyme genes after 0 h, 6 h, and 24 h JA treatment.

To investigate the potential involvement of *TkMYB7* in natural rubber (NR) biosynthesis, cis-regulatory element analysis was performed on the promoter regions of genes in the mevalonate (MVA) pathway—the core metabolic route for NR synthesis. Visualization of promoter architecture (Figure 5A) revealed that all genes in the pathway possessed MYB transcription factor (TF) target sites, specifically G-BOX elements, which are typically associated with light-responsive regulation. Furthermore, most promoters were enriched with stress- and hormone-responsive cis-elements, including motifs associated with methyl jasmonate (MeJA) responsiveness (TGACG-motif, CGTCA-motif) and low-temperature responses (LTR). These findings suggest functional diversity and regulatory specificity among MVA pathway genes.

To explore the broader jasmonate (JA)-mediated regulation of the TKS MVA pathway, expression profiles of key enzyme-encoding genes were assessed following JA treatment. The results (Figure 5B) showed that within the hydroxymethylglutaryl-CoA reductase (HMGR) family, *HMGR3*, *HMGR2*, and *HMGR8* exhibited the highest transcript abundances after JA induction. Similarly, in the acetoacetyl-CoA thiolase (ACAT) family, *ACAT5* and *ACAT7* displayed significantly higher expression levels compared with other family members. These data underscore the selective activation of specific paralogs within the MVA pathway under JA signaling, highlighting potential regulatory nodes for NR biosynthesis. We focused on HMGR and ACAT families due to their pivotal roles in controlling metabolic flux: HMGR as the recognized rate-limiting enzyme and ACAT as the catalyst for the first committed step of the pathway. Among the highly JA-responsive ACAT members, *TkACAT5* exhibited the most pronounced and consistent upregulation. Therefore, *TkACAT5* was selected as the primary target to investigate whether *TkMYB7* directly regulates the gateway of the rubber biosynthesis pathway.

### 2.6. Y1H Experiment Proves That TkMYB7 Can Regulate TkACAT5 in the MVA Pathway

**Figure 6:**
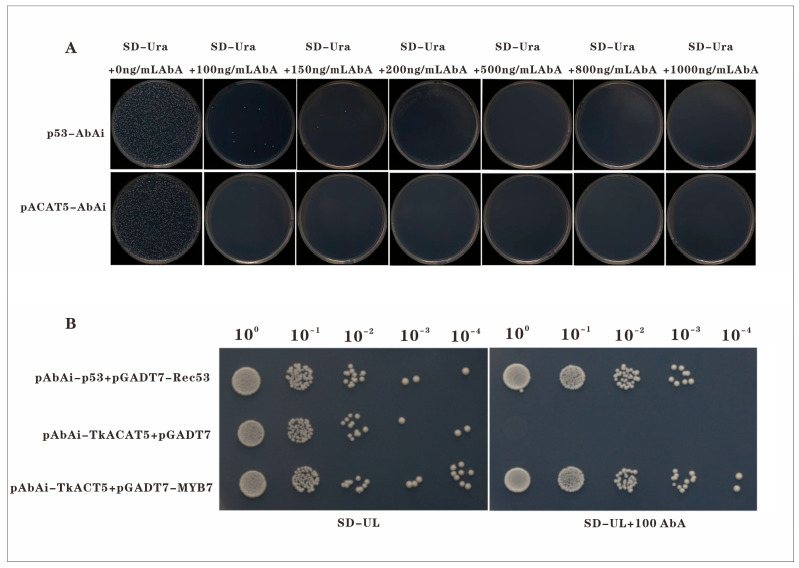
Yeast one-hybrid assay of TkMYB7 and TkACAT5: (**A**) Screening for the minimum AbA concentration inhibiting bait strain autoactivation. (**B**) Spot assay validating the interaction between TkMYB7 and the TkACAT5 promoter. Positive control: pAbAi-p53 + pGADT7-Rec53; Negative control: pAbAi-TkACAT5 + empty pGADT7. Scale bars = 1 cm.

In the initial stage of the yeast one-hybrid (Y1H) assay, the optimal concentration of Aureobasidin A (AbA) required to suppress autoactivation of the bait strain was determined. The bait construct pAbAi–TkACAT5 was transformed into the bait strain, serially diluted, and plated on selective medium containing different AbA concentrations (Figure 6A). Autoactivation of the pAbAi–TkACAT5 strain was effectively inhibited at 100 ng/mL AbA, which was subsequently used in the interaction assays.

Yeast one-hybrid spot analysis (Figure 6B) revealed that on selective medium lacking AbA (SD/–Ura/–Leu), colonies of the positive control (pAbAi–p53 + pGADT7–Rec53), negative control (pAbAi–TkACAT5 + empty pGADT7), and experimental group (pAbAi–TkACAT5 + pGADT7–TkMYB7) all grew. In contrast, on medium supplemented with 100 ng/mL AbA, growth was observed in the positive control and experimental group, whereas the negative control showed no growth.

These findings indicate that TkMYB7 specifically interacts with the promoter of *TkACAT5*, a key enzyme-encoding gene in the natural rubber biosynthesis pathway. The Y1H evidence confirms that TkMYB7 can directly bind to the *TkACAT5* promoter, validating prior bioinformatic predictions and providing molecular support for the role of TkMYB7 in jasmonate (JA)-mediated transcriptional regulation of NR biosynthesis.

### 2.7. Stable Inheritance of TkMYB7 in Arabidopsis thaliana

To investigate the role of TkMYB7 in plant growth and development, stable overexpression lines of *Arabidopsis thaliana* were established and confirmed (Supplementary Figure S1). Seed germination and root growth phenotypes were compared between the transgenic lines and wild-type (WT) plants in the absence of exogenous jasmonate (JA).

**Figure 7:**
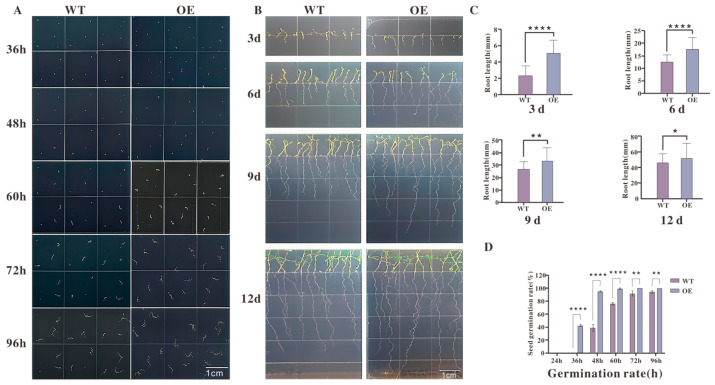
Phenotypic analysis of seed germination and root growth in transgenic Arabidopsis lines: (**A**) Representative images of seed germination at 36 h, 48 h, 60 h, 72 h, and 96 h after sowing. OE plants germinated earlier and had a higher proportion of germinated seeds at each time point compared with WT. (**B**) Root elongation of WT and OE seedlings at 3, 6, 9, and 12 days after germination. OE plants exhibited more rapid root growth throughout the observation period. (**C**) Quantitative measurements of root length at each time point, showing significantly greater root lengths in OE plants relative to WT (**** *p* < 0.0001 at 3 d; ** *p* < 0.01 at 6 d; ** *p* < 0.01 at 9 d; * *p* < 0.05 at 12 d). (**D**) Germination rates calculated over time, indicating significantly higher germination percentages in OE plants than WT between 48 h and 72 h (**** *p* < 0.0001). Data are presented as mean ± standard error (n = 3 biological replicates). Statistical significance was determined by Student’s *t*-test. Scale bars = 1 cm.

The results (Figure 7A) showed that transgenic seeds initiated germination earlier, with visible germination at 36 h and a greater number of germinated seeds between 60 and 72 h. Quantitative analysis of germination rates (Figure 7D) revealed that transgenic lines consistently exhibited significantly higher germination rates than WT between 48 and 72 h. Root length measurements (Figure 7B) indicated that both WT and transgenic seedlings began root elongation by day 3; however, by day 6, transgenic roots were significantly longer than those of WT plants.

Under standard growth conditions without hormonal treatment, transgenic Arabidopsis lines overexpressing TkMYB7 displayed a consistently significant phenotype characterized by accelerated seed germination and enhanced root growth when compared to the wild-type (Figure 7).

## 3. Discussion

With the continuous advancement of biotechnology, whole-genome sequencing of plant species has become increasingly common. The MYB transcription factor family has been identified in numerous plants, such as *Arabidopsis thaliana* [44], *grape* [45], and *rice* [46]. However, research on this family remains limited in *Taraxacum kok-saghyz* Rodin (TKS), which is an emerging alternative source of natural rubber. Therefore, this study aims to conduct a genome-wide identification of the MYB family in TKS, investigate its role in the jasmonic acid (JA) signaling pathway, and identify potential key genes.

In our previous work, transcriptome sequencing of wild-type TKS treated with methyl jasmonate (MeJA) for 0, 6, and 24 h suggested that JA may regulate natural rubber biosynthesis and other secondary metabolites in TKS by interacting with transcription factors associated with rubber synthesis [47]. Building on these findings, the present study seeks to identify the MYB family in TKS using its whole-genome database, screen for genes responsive to both JA and ethylene using transcriptome data, and select a target gene of significance through tissue-specific qRT-PCR expression analysis. Ultimately, this research aims to elucidate the regulatory mechanisms underlying natural rubber (NR) biosynthesis.

In this study, we conducted a comprehensive identification of MYB family proteins in TKS using a local BLAST (https://blast.ncbi.nlm.nih.gov/Blast.cgi; accessed 9 August 2024) analysis with the MYB protein sequences from *Arabidopsis thaliana* as queries to search for proteins containing the R2R3-MYB domain. Based on the whole-genome data of TKS published in 2017 [4], a total of 268 MYB family members were identified. Phylogenetic analysis was subsequently performed on the MYB families of TKS and *Arabidopsis thaliana*. The resulting phylogenetic tree demonstrated that the 394 MYB proteins from these two species could be classified into four major clades (Figure 1). Most TkMYB and AtMYB proteins clustered within the same subgroups, indicating close genetic relationships. Notably, TkMYB7 was grouped together with AtMYB77 and AtMYB44 within the same subclade, suggesting a close evolutionary relationship between TkMYB7 and its *Arabidopsis* homologs.

By integrating transcriptome data in response to jasmonic acid and ethylene, we identified 10 candidate MYB genes exhibiting consistent upregulation. The sequences of these MYBs showed high similarity to those annotated in the 2017 *Taraxacum koksaghyz* (TKS) genome database [4]. Notably, *TkMYB7* was among these upregulated genes, drawing our particular research interest. Subsequently, we conducted comprehensive analyses of the phylogenetic relationships, protein architectures, gene structures, and physicochemical properties of these 10 MYB genes. Our integrated bioinformatic analysis systematically characterized the fundamental features of these MYB genes and, more importantly, established insightful connections between their evolutionary history, molecular structures, and potential functions. These efforts laid a solid foundation for further functional investigations.

Based on these findings, we extracted RNA from various tissues of TKS, reverse-transcribed it into cDNA, and performed expression pattern analysis. The results revealed notably high expression of *TkMYB7* in the roots. Given that NR is predominantly synthesized in the roots of TKS, we applied jasmonic acid and ethylene treatments and conducted transcriptome analysis specifically on root tissues. All 10 MYB genes showed upregulated expression under both hormone treatments (Figure 2), consistent with the observed tissue-specific expression profiles. These results confirmed *TkMYB7* as the target gene for subsequent functional studies. To elucidate its subcellular localization, we performed predictive analysis followed by experimental validation, which demonstrated that *TkMYB7* functions in the nucleus.

Recent studies have increasingly demonstrated that MYB genes play extensive roles in plant responses to abiotic stresses by regulating downstream target genes. For instance, in apple guard cells, *MdMYB44*-like directly binds to the MBS element in the promoter of *MdPP2CA*, repressing its transcription. This repression is further enhanced through interaction with the ABA receptor protein *MdPYL8* [48]. In *Arabidopsis*, *AtMYB103* acts as a positive regulator of aluminum tolerance by activating the transcription of *TRICHOME BIREFRINGENCE-LIKE 27 (TBL27)*, a gene involved in xyloglucan O-acetylation. This modification reduces aluminum binding to the cell wall, thereby enhancing aluminum tolerance in transgenic plants [49]. These findings indicate that MYB transcription factors participate in the regulation of plant physiological processes by integrating into signaling pathways through direct DNA binding, ultimately modulating the synthesis and release of metabolites. Furthermore, treatment with methyl jasmonate (MeJA) upregulates the expression of multiple MYB genes [18], supporting the involvement of MYB transcription factors in jasmonic acid (JA) signaling. This observation indirectly suggests that natural rubber biosynthesis in TKS may be associated with MYB-mediated JA signaling pathways.

In TKS, genes of the ACAT family play a crucial role in the natural rubber biosynthesis pathway, primarily within the mevalonate (MVA) pathway. Although ACAT family genes are not directly involved in the polymerization and elongation of rubber chains, they catalyze the conversion of acetyl-CoA to acetoacetyl-CoA [50], which is a vital reaction for producing the precursor of rubber monomers.

In this study, we screened key enzyme genes of the MVA pathway from the TKS whole-genome data and selected acetyl-CoA acetyltransferase (*TkACAT5*) as a candidate for interaction validation based on its jasmonic acid-responsive expression patterns (Figure 5B). Furthermore, expression analysis revealed that *TkMYB7* was also upregulated in response to jasmonic acid (JA) (Figure 2A), suggesting that both genes may cooperatively regulate natural rubber biosynthesis under JA induction. These findings provide preliminary evidence for a functional interaction between *TkMYB7* and *TkACAT5*. This hypothesis was subsequently confirmed through a yeast one-hybrid assay (Figure 6B), validating the regulatory relationship between *TkMYB7* and the promoter of *TkACAT5*.

The yeast one-hybrid (Y1H) assay unequivocally demonstrated the direct binding of *TkMYB7* to the promoter region of *TkACAT5*, providing critical molecular evidence that links upstream jasmonate (JA) and ethylene signaling to the downstream rubber biosynthetic pathway. This experimentally validated interaction not only corroborates prior bioinformatic predictions but also firmly establishes a functional transcription factor–target gene relationship, which likely represents a core element within the JA-responsive regulatory module governing natural rubber biosynthesis. Given that ACAT catalyzes the first committed step in the mevalonate (MVA) pathway, the direct transcriptional regulation of *TkACAT5* by *TkMYB7* positions this transcription factor as a strategic regulatory node at the gateway of rubber precursor synthesis. This regulatory arrangement aligns with established paradigms in other rubber-producing species, where JA-responsive transcription factors modulate key enzymes in the isoprenoid biosynthetic pathway [16].

A definitive genetic dissection of its precise position within the JA/ET signaling hierarchy, utilizing hormone receptor mutants, represents the logical and essential next step to build upon the mechanistic foundation established in this study. Likewise, directly evaluating the impact of *TkMYB7* on stress tolerance and rubber content under abiotic stresses in its native TKS system, via CRISPR/Cas9-mediated gene editing or overexpression, will be a priority of our future work to fully elucidate its multifunctional roles.

Furthermore, the potential functions of MYB family genes in plants have attracted increasing research interest. Previous studies have demonstrated that *OsMYB2* in rice is induced under salt, cold, and dehydration stresses [51]. Overexpression of *PagMYB151* in poplar enhances salt tolerance by promoting proline accumulation [52], while *AtMYB41* in *Arabidopsis* improves drought resistance through an ABA-mediated pathway [53]. In line with these findings, the present study successfully introduced and stably expressed *TkMYB7* in *Arabidopsis*, as confirmed by screening T3 transgenic seeds and conducting DNA-level verification. Germination assays comparing transgenic and wild-type seeds revealed a significantly higher germination rate in the transgenic lines, suggesting that MYB family genes play a notable role in promoting seed germination.

The heterologous overexpression of *TkMYB7* in *Arabidopsis thaliana*—a system devoid of the metabolic complexity of rubber biosynthesis—clearly established that this transcription factor intrinsically accelerates seed germination and enhances root development under standard growth conditions (Figure 7). This phenotype provides direct functional validation, confirming *TkMYB7* as a potent regulator of core plant developmental processes. The observation of this phenotype without exogenous hormone application indicates that the constitutive overexpression of *TkMYB7* is sufficient to recapitulate developmental programs, potentially by mimicking an activated transcriptional state downstream of hormonal signals.

Critically, this functional evidence does not stand in isolation. Instead, it forms a coherent and self-reinforcing narrative when integrated with our other key discoveries in TKS: (1) The root-predominant expression of *TkMYB7* (Figure 4A) confirms the biological relevance of the observed *Arabidopsis* root phenotype to its native context. (2) Its direct transcriptional activation of *TkACAT5* (Figure 6), encoding the enzyme that catalyzes the first committed step of the MVA pathway, firmly links *TkMYB7* to the rubber biosynthetic machinery. (3) Its specific induction by both jasmonate and ethylene (Figure 2) positions *TkMYB7* precisely within the hormonal framework known to promote natural rubber biosynthesis.

Taken together, the enhanced root growth observed in *Arabidopsis* can be regarded as a strong indicator of *TkMYB7*’s inherent ability to drive organ development—a capacity that, in *TKS*, is likely harnessed to optimize both root system architecture (the “factory”) and the output of the rubber biosynthetic pathway (the “product”). The integrative model emerging from our data places *TkMYB7* as a central regulatory node that channels JA/ethylene signals into coordinated developmental and metabolic outcomes. Additionally, its induction by these stress-associated hormones suggests a potential role in abiotic stress adaptation, highlighting an attractive avenue for future research.

To further delineate its position within the JA/ET signaling hierarchy, a genetic dissection using hormone receptor mutants represents an essential next step toward deepening the mechanistic framework established in this study. Moreover, evaluating the impact of *TkMYB7* on stress tolerance and rubber yield under abiotic stress conditions in *TKS*, through CRISPR/Cas9-mediated gene editing or targeted overexpression, will be a priority in our future work. These investigations will be instrumental in fully elucidating the multifunctional roles of *TkMYB7*.

## 4. Materials and Methods

### 4.1. Plant Materials and Treatments

TKS plants were collected from the Ili region of the Tekes River basin in Xinjiang, China, and were successfully propagated and cultivated in our laboratory. TKS seedlings were grown under standardized conditions (potting mix: vermiculite = 1:1, temperature: 25 °C, light/dark cycle: 16 h/8 h) until they reached the flowering stage. Following this, four different tissue types—root, leaf, flower, and scape—were collected in foil packages, ensuring three biological replicates were obtained for each tissue type. The samples were then flash-frozen in liquid nitrogen. Total RNA was extracted from the TKS roots using the Universal Plant Total RNA Extraction Kit (TransGen Biotech, Beijing, China) according to the manufacturer’s instructions. Residual genomic DNA was removed using DNase I treatment, and RNA integrity was assessed via electrophoresis on a 2% agarose gel. cDNA was synthesized from 1 µg of total RNA using the EasyScript^®^ One-Step gDNA Removal and cDNA Synthesis Kit (TransGen Biotech, Beijing, China) for subsequent gene cloning purposes. The remaining tissue samples were stored at −80 °C for future analyses.

### 4.2. Identification and Characterization of MYB Genes in TKS

The protein sequences of 126 known *Arabidopsis thaliana* MYB family members were retrieved from the TAIR database (https://www.arabidopsis.org) [54]. To comprehensively identify homologous MYB proteins in TKS, two independent search strategies were applied to the publicly available TKS whole-genome dataset (BioProject: PRJCA000437) from the Genome Warehouse (GWH; http://bigd.big.ac.cn/gwh, accessed on 18 April 2024) [4]. BLASTP Search: The *Arabidopsis* MYB sequences were used as queries for a local BLASTP search against the TKS proteome. An E-value threshold of <1 × 10^−5^ and a minimum sequence similarity of >50% were employed as cutoffs, yielding 255 candidate MYB proteins in TKS. HMMER Search: The hidden Markov model (HMM) profile for the MYB DNA-binding domain (PF00249) [55] was obtained from the Pfam database. The profile was used to scan the TKS proteome via HMMER [56], resulting in the identification of 268 candidate proteins containing the conserved MYB domain. Candidate lists obtained from both approaches were merged, and redundant sequences were removed, producing a final non-redundant set of 268 MYB proteins in TKS.

The presence of conserved domains in all candidates was rigorously verified using the NCBI Conserved Domain Database (CDD) tool (http://www.ncbi.nlm.nih.gov/Structure/cdd/wrpsb.cgi; accessed on 16 August 2024). Proteins were classified as R2R3-MYB if they contained a MYB domain with two R2/R3 repeats located at the N-terminus and lacked additional domains. All 268 proteins fulfilled these criteria and were conclusively assigned to the R2R3-MYB subfamily [57]. For phylogenetic analysis, multiple sequence alignment was performed between the 126 *Arabidopsis* MYB proteins and the 268 identified *TkMYB* sequences. A neighbor-joining tree was constructed with 1000 bootstrap replicates [58] to assess branch reliability. The resulting tree was annotated and visualized using the Interactive Tree Of Life (iTOL) website (https://itol.embl.de/; accessed on 18 April 2024) [59].

### 4.3. Transcriptome Data Analysis

The expression patterns of *TkMYB* genes were analyzed using Fragments Per Kilobase of transcript per Million mapped reads (FPKM) values, which were then log2-transformed [60]. Heatmaps of the FPKM data were generated using TBtools software (Tbtools v2.363) [56]. Transcriptome data obtained from *MeJA* and ethylene (ET)-treated TKS samples were analyzed to identify genes commonly upregulated by both treatments.

A phylogenetic tree was constructed for the selected genes, and exon–intron distribution information for MYB genes was extracted from the GFF annotation file. Conserved motifs of MYB family proteins were analyzed using the MEME Suite (https://web.mit.edu/meme/current/share/doc/overview.html; accessed on 10 August 2024). All analysis files were integrated and visualized using TBtools. The physicochemical properties of the selected *TkMYB* proteins were evaluated using ExPASy (https://web.expasy.org/compute_pi/; accessed on 18 August 2024). Subcellular localization predictions were made using Plant-mPLoc (http://www.csbio.sjtu.edu.cn/) (Table 1).

### 4.4. Subcellular Localization Analysis of TkMYB Proteins

The tissue expression patterns of the selected *TkMYB* genes were analyzed. RNA was extracted from four tissue types (root, leaf, flower, and scape) and reverse-transcribed to cDNA, following the protocol outlined in Section 2.1 (primers listed in Table S1). Quantitative PCR (qPCR) was performed using Ultra SYBR Mixture (Low ROX) (CWBIO, Beijing, China) on an IQ5 Real-Time PCR system (Bio-Rad, Hercules, CA, USA). β-actin served as the internal reference gene, and gene expression values were normalized using the 2^−ΔΔCt^ method. In conjunction with transcriptome analysis, *TkMYB7* was identified as the target gene for subsequent studies. The *TkMYB7* coding sequence (CDS) was successfully cloned and ligated into the pMD-19T vector, serving as a universal template for further experiments (sequences and primers provided in Tables S2 and S3).

The coding sequence of TkMYB7 was amplified with specific primers (Table S4) and cloned into the pAN580-GFP vector using the ClonExpress One Step Cloning Kit (Vazyme), resulting in the pAN580-TkMYB7-GFP construct. The construct was verified by sequencing. For subcellular localization, the pAN580-TkMYB7-GFP plasmid and a chloroplast marker (pAN580-Chlorophyll-RFP) were co-transfected into Arabidopsis protoplasts using a standard polyethylene glycol (PEG) method. After 14–18 h, fluorescence signals were observed using a confocal microscope (Nikon N-SIM S) [61].

### 4.5. Screening for Downstream Genes of TkMYB7

Based on preliminary jasmonic acid (JA)-treated transcriptome data generated in our laboratory, differentially expressed genes (DEGs) related to natural rubber (NR) synthesis were screened and identified using the Majorbio Cloud Platform online transcriptome analysis tool. The screening criteria applied were a *p*-value ≤ 0.05 and |log2(fold change)| ≥ 1. DEGs were further validated through quantitative PCR (qPCR) analysis of *TKS* samples treated with exogenous JA at various time points. The promoters of the DEGs associated with NR synthesis were analyzed using the PlantCARE online database to predict the presence of G-box elements, which serve as binding sites for MYB family transcription factors (TFs). Ultimately, the most significantly differentially expressed gene identified was acetyl CoA acetyltransferase *TkACAT5* (Gene ID listed in Table S5).

### 4.6. Yeast One-Hybrid Bait Vector Construction and Validation (Aiming to Eliminate False Positives)

The bait vector pAbAi-ACAT5, containing tandem repeats of the G-box element from the *TkACAT5* promoter, was commercially synthesized (Nuowa Biology). The prey vector pGADT7-*TkMYB7* was constructed as described in Section 4.4. The bait strain was generated by transforming linearized pAbAi-ACAT5 into *Y1H* Gold yeast cells. The minimum inhibitory concentration of Aureobasidin A (AbA) for the bait strain was determined to be 100 ng/mL. For the interaction assay, the pGADT7-*TkMYB7* plasmid was transformed into the bait strain, which was then plated on SD/-Leu-Ura medium supplemented with 100 ng/mL AbA.

The prey vector pGADT7-TkMYB7 recombinant plasmid was then transformed into the Bait strain. The positive control used was pAbAi-p53 + pGADT7-Rec53, while the negative control consisted of pAbAi-TkACAT5 + empty pGADT7. Transformants were plated on SD/-Ura/-Leu medium containing the predetermined minimal inhibitory concentration of AbA and incubated at 30 °C for 3–4 days to observe yeast growth.

### 4.7. Phenotypic Identification and Statistical Analysis of Seed Germination and Root Growth in Transgenic Lines

Transgenic *Arabidopsis thaliana* lines were generated using the floral dip method. Harvested T1 seeds were screened on 1/2 strength MS plates containing kanamycin and streptomycin (50 mg·mL^−1^). The phenotypes of transgenic plants were verified in at least three independent lines, and homozygous T3 lines were identified based on kanamycin resistance. Transgenic *Arabidopsis thaliana* lines were generated using the floral dip method. Briefly, the pCAMBIA-1300-eGFP-TkMYB7 construct was introduced into *Agrobacterium tumefaciens* GV3101, which was then used to transform wild-type (Col-0) plants. T1 seeds were selected on 1/2 MS medium containing kanamycin (50 mg/L), and were used for subsequent phenotypic analysis [62].

Treated plants were kept in the dark for 24 h before returning to normal growth conditions (to improve efficiency, the infiltration can be repeated after one week). Seeds were harvested after approximately one month of normal growth and subjected to screening. The growth conditions for Arabidopsis were maintained at 22 °C, with a light/dark cycle of 16 h/8 h and humidity at 40%. Seed germination assays were conducted with both transgenic and wild-type (WT) Arabidopsis seeds under identical conditions. Germination rates were recorded, and root lengths were measured using a calibrated ruler.

## 5. Conclusions

In this study, we present a functional characterization of TkMYB7, a JA- and ethylene-responsive R2R3-MYB transcription factor in *Taraxacum kok-saghyz*. Our key findings demonstrate that TkMYB7 is a nuclear-localized protein that acts as a direct positive regulator of the natural rubber biosynthetic pathway. This conclusion is robustly supported by the following evidence: (1) TkMYB7 exhibits root-predominant expression and is significantly upregulated by JA and ethylene treatments; (2) it directly binds to the promoter of TkACAT5, a gene encoding a pivotal enzyme in the mevalonate (MVA) pathway; and (3) its heterologous overexpression in Arabidopsis enhances root growth, underscoring its role in modulating developmental processes.

Therefore, we propose a model wherein TkMYB7 serves as a crucial transcriptional regulator that integrates JA and ethylene signaling to modulate natural rubber biosynthesis in TKS, likely by activating the expression of TkACAT5 to fuel the MVA pathway. Our work identifies TkMYB7 as a central component of the transcriptional network governing rubber production and provides a solid theoretical foundation and a promising genetic target for future breeding strategies aimed at enhancing rubber yield in TKS.

Future work will focus on directly validating the impact of *TkMYB7* on rubber content in its native system using CRISPR/Cas9-mediated gene editing and overexpression, and further dissecting its upstream regulatory mechanisms and downstream target gene networks.

## Figures and Tables

**Table 1:** Physicochemical properties and subcellular localization prediction of the 10 identified TKS MYB family members.

Gene Name	ID (2017) [4]	Amino Acid	Molecular Weight (kDa)	Theoretical pI	Instability Index	GRAVY	Predicted Location
TkMYB7	evm.model.utg478.9	233	26.06	7.68	57.59	−0.745	Nucleus
TkMYB224	evm.model.utg1206.14	226	25.9	6.18	50.39	−0.803	Nucleus
TkMYB206	evm.model.utg25688.5	312	35.42	5.77	47.79	−0.775	Nucleus
TkMYB196	evm.model.utg20126.1	274	30.75	5.94	48.42	−0.87	Nucleus
TkMYB228	evm.model.utg24099.1	262	28.98	7.03	46.51	−0.59	Nucleus
TkMYB19	evm.model.utg22022.2	313	34.92	9.14	56.04	−0.795	Nucleus
TkMYB231	evm.model.utg1784.24	268	31.04	6.83	52.52	−0.848	Nucleus
TkMYB208	evm.model.utg3625.1	268	31.06	6.83	54.52	−0.858	Nucleus
TkMYB51	evm.model.utg21778.3	207	24.35	7.70	65.70	−1.114	Nucleus
TkMYB28	evm.model.utg24885.2	400	45.05	9.97	63.54	−0.578	Nucleus

ID (2017): identity code of *TkMYBs* in the 2017 version of a TKS genome.

## Data Availability

The primers involved in this research are all in the Tables S1–S7, and the templates synthesized by the primers are all cDNA. The TKS genome and nucleotide sequences were deposited in the Genome Warehouse (GWH; http://bigd.big.ac.cn/gwh/, accessed on 18 April 2024) under the accession number PRJCA000437.

## Supplementary Materials

The following supporting information can be downloaded at: https://www.mdpi.com/article/10.3390/plants14213323/s1; Table S1: Primers used for qRT-PCR analysis; Table S2: Coding sequence (CDS) of *TkMYB7*; Table S3: Primers used for amplifying *TkMYB7*; Table S4: Primers used for subcellular localization vector construction; Table S5: Gene IDs of key enzymes in the MVA pathway; Table S6: Primers used for yeast one-hybrid vector construction; Table S7: Primers used for overexpression vector construction; Figure S1: Molecular validation of *TkMYB7* overexpression in transgenic lines.
