# Supplementary material for: TkMYB7 Coordinates Jasmonate and Ethylene Signaling to Regulate Natural Rubber Biosynthesis in Taraxacum kok-saghyz
Source: Plants (Basel). 2025 Oct 30;14(21):3323. doi: 10.3390/plants14213323 (PMC12608830; doi:10.3390/plants14213323)
Supplement: Supplementary file 1 [file plants-14-03323-s001.zip › Figure S1.pdf]

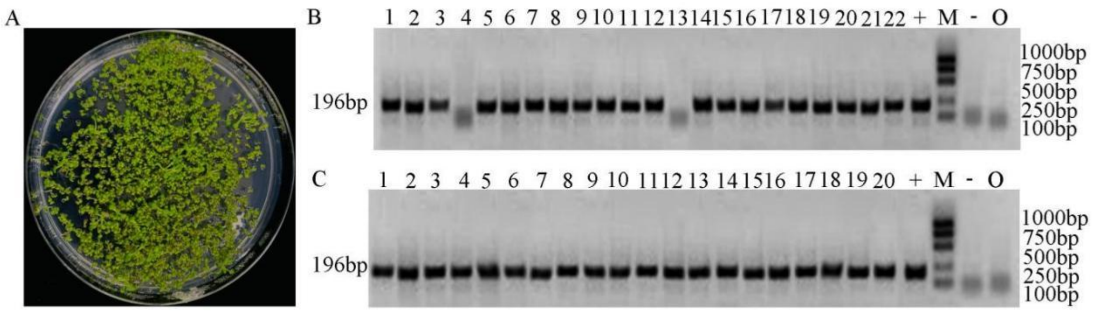

Figure S1 Seed germination and DNA level identification of transgenic Arabidopsis thaliana. (A) Transgenic Arabidopsis grows on resistant plates (kan/sm=50mg·mL−1). (B) DNA identification of T1 transgenic Arabidopsis thaliana. (C) DNA identification of T3 transgenic Arabidopsis thaliana.
